# Identification of kidney-related medications using AI from self-captured pill images

**DOI:** 10.1080/0886022X.2024.2402075

**Published:** 2024-09-11

**Authors:** Mohammad S. Sheikh, Benjamin Dreesman, Erin F. Barreto, Charat Thongprayoon, Jing Miao, Supawadee Suppadungsuk, Michael A. Mao, Fawad Qureshi, Justin H. Pham, Iasmina M. Craici, Kianoush B. Kashani, Wisit Cheungpasitporn

**Affiliations:** a Division of Nephrology and Hypertension, Department of Medicine, Mayo Clinic, Rochester, MN, USA; b Department of Pharmacy, Mayo Clinic, Rochester, MN, USA; c Chakri Naruebodindra Medical Institute, Mahidol University, Samut Prakan, Thailand; d Division of Nephrology and Hypertension, Department of Medicine, Mayo Clinic, Jacksonville, FL, USA; e Division of Pulmonary and Critical Care Medicine, Department of Medicine, Mayo Clinic, Rochester, MN, USA

**Keywords:** AI, kidney, nephrology, digital health, medication identification, pill image analysis

## Abstract

**Introduction:**

ChatGPT, a state-of-the-art large language model, has shown potential in analyzing images and providing accurate information. This study aimed to explore ChatGPT-4 as a tool for identifying commonly prescribed nephrology medications across different versions and testing dates.

**Methods:**

25 nephrology medications were obtained from an institutional pharmacy. High-quality images of each medication were captured using an iPhone 13 Pro Max and uploaded to ChatGPT-4 with the query, ‘What is this medication?’ The accuracy of ChatGPT-4’s responses was assessed for medication name, dosage, and imprint. The process was repeated after 2 weeks to evaluate consistency across different versions, including GPT-4, GPT-4 Legacy, and GPT-4.Ø.

**Results:**

ChatGPT-4 correctly identified 22 out of 25 (88%) medications across all versions. However, it misidentified Hydrochlorothiazide, Nifedipine, and Spironolactone due to misreading imprints. For instance, Nifedipine ER 90 mg was mistaken for Metformin Hydrochloride ER 500 mg because ‘NF 06’ was misread as ‘NF 05’. Hydrochlorothiazide 50 mg was confused with the 25 mg version due to imprint errors, and Spironolactone 25 mg was misidentified as Naproxen Sodium or Diclofenac Sodium. Despite these errors, ChatGPT-4 showed 100% consistency when retested, correcting misidentifications after receiving feedback on the correct imprints.

**Conclusion:**

ChatGPT-4 shows strong potential in identifying nephrology medications from self-captured images, though challenges with difficult-to-read imprints remain. Providing feedback improved accuracy, suggesting ChatGPT-4 could be a valuable tool in digital health for medication identification. Future research should enhance the model’s ability to distinguish similar imprints and explore broader integration into digital health platforms.

## Introduction

Patients in the modern age are more prone to medication errors due to more drugs with advances in drug research, coexistence of multiple comorbidities, and resulting complex drug regimens. One of the major contributing factors to medication error is polypharmacy, which is defined as receiving more than five drugs [1–4]. The prevalence of polypharmacy significantly increased from 8.2% in 1990 to 17.1% in 2018 [1–4]. Polypharmacy has had negative associations with patient safety, including medication errors, nonadherence, adverse drug events, drug-drug interaction, hospitalizations, and mortality [4–10]. Patients with chronic kidney disease (CKD) represent a cohort of the population at the highest polypharmacy risks due to CKD-associated multiple comorbidities, increased daily pill burden, and the complexity of medication management [10–14]. Prior studies have substantiated that polypharmacy was identified as a significant risk factor for the high reported prevalence of medical errors at 68% in nephrology [10]. An effective first strategy to manage polypharmacy and reduce medication errors for patients’ safety involves medication reconciliation [15–17]. Medication reconciliation can be challenging, however, as patients often have several providers that prescribe medications that may not be captured in a single medical record system, such as multi-department outpatient clinics, after-hospital discharge, over-the-counter medications, and complementary medicine or other supplements. Medication reconciliation can also be challenging for doctors and pharmacists when patients only provide pills without medical labels [18]. Patient recalling their home medications can also be prone to errors due to look-alike or sounds-alike (LASA) names [19]. The use of artificial intelligence (AI) may help in this challenging but important aspect of medical care, as AI has already been implemented to assist in drug counseling [20] and identifying drug-drug interactions [21, 22].

ChatGPT, a large language model developed by OpenAI [23], has been increasingly integrated widely into healthcare. ChatGPT-4 displays more human-like versatility in conversing coherently on open-ended topics [24–27]. Since its release in 2022, ChatGPT has shown aptitude in assimilating both textual and visual inputs from complex prompts to produce responsive narratives. Several potential uses for ChatGPT as a healthcare tool include refining the healthcare provider’s decision-making process, providing patient information and education, clinical documentation, and research. In the context of nephrology, previous studies have demonstrated that ChatGPT could assist in providing personalized renal nutritional dietary advice and tailored CKD patient education [28–31]. Assessments across medical disciplines have also confirmed its ability to analyze images and radiographic studies to generate accurate diagnostic and management recommendations [32]. However, ChatGPT −4’s reliability in identifying pharmacotherapy pertinent to specialized fields like nephrology remains unexplored.

This study aims to bridge this knowledge gap by exploring ChatGPT −4’s accuracy in identifying medications commonly used in nephrology practice *via* self-captured images. Our primary objective was to evaluate the model’s performance in drug identification. The secondary aims included assessing the consistency of ChatGPT −4’s responses and its ability to integrate feedback to improve performance. Demonstrating ChatGPT −4’s capabilities in this focused task could pave the way for larger research efforts on integrating AI to improve nephrology practice.

## Methods

This study was conducted at the Mayo Clinic in Rochester, Minnesota, in January 2024. The goal was to evaluate the ability of ChatGPT-4 to identify kidney-related medications using high-quality images of the pills. We carefully selected 25 oral medications that are frequently prescribed in nephrology, based on expert recommendations from both nephrologists and pharmacists. These medications included angiotensin-converting enzyme inhibitors (lisinopril, enalapril), angiotensin II receptor blockers (losartan, candesartan), thiazide diuretics (chlorthalidone, hydrochlorothiazide), loop diuretics (furosemide), calcium-channel blockers (amlodipine, nifedipine), beta-blockers (carvedilol, metoprolol), mineralocorticoid receptor antagonists (spironolactone), combination pills (lisinopril/hydrochlorothiazide), hydralazine, and other medications commonly used in managing chronic kidney disease (aspirin, allopurinol, colchicine, gabapentin, ketorolac, lithium, omeprazole, sotalol, sulfamethoxazole/trimethoprim, trazodone).

To prepare the images, two identical sample pills of each selected medication were obtained from the central pharmacy. Each pair of pills was then placed side by side on a neutral grey background, with one pill showing the front and the other showing the back. This setup was designed to capture all possible identifying marks on the pills. The images were captured using an iPhone 13 Pro Max in a well-lit environment, with the camera positioned approximately 15.24 centimeters (about 6 inches) above the pills. This distance ensured that the entire pill surface was within the frame, while still capturing fine details. The camera’s maximum resolution of 4032 × 3024 pixels was used, which provided a high pixel density of at least 300 pixels per inch (PPI), ensuring that the imprints and other distinguishing features on each medication were clearly visible.

### ChatGPT-4 queries

The procedure commenced with providing ChatGPT-4 with a specific instruction to employ its browser capability to ascertain the identity of the medication. This was done using the prompt: ‘use browser to identify the answer of the following next prompt’. Following this directive, de-identified pill images were uploaded to ChatGPT-4. Each image was then queried with the subsequent text prompt: ‘What is this medication?’ Importantly, no additional information concerning the medication’s group, clinical indication, or imprint code was provided alongside the queries.

ChatGPT-4’s ability to identify the medication name, dosage, and pill imprint by image was reviewed and assessed for accuracy by study investigators. If ChatGPT-4 did not accurately identify the medication due to misreading the pill imprint, it was provided with the correct feedback on the imprint and asked to generate another response using the following prompt: ‘The correct imprint for this medication is XXX; what medication is this?’. The entire image query process was repeated across multiple versions of ChatGPT-4, including GPT-4 (12/12/23, 12/30/23), GPT-4 Legacy (08/22/24, 08/29/24), and GPT-4.Ø (08/22/24, 08/29/24), to assess consistency and reliability of the AI responses over time.

### Statistical analysis

The primary outcome measure of this study was the accuracy of ChatGPT-4 in identifying the medications based on the provided images. Accuracy was calculated as the percentage of correctly identified medications out of the total number of medication images tested. A correctly identified medication was defined as an instance where ChatGPT-4 accurately identified the medication name, dosage, and imprint. The total number of medication images tested was 25, with each medication being tested once in the initial round and once in the repeat round after a 2-week interval.

Secondary outcomes included the consistency of ChatGPT-4’s responses between the initial and repeat rounds, as well as the model’s ability to improve its accuracy when provided with corrective feedback on misidentified medications. Consistency was assessed by comparing the responses between the two rounds of testing, while the effect of corrective feedback was evaluated by re-testing the misidentified medications after providing the correct imprint information.

The focus was on providing descriptive statistics to summarize ChatGPT-4’s performance in identifying medications from images.

## Results

ChatGPT-4’s response to queries for medication identification from images is shown in Table 1 and Supplementary Material. Across different versions and testing dates, including GPT-4 (12/12/23, 12/30/23), GPT-4 Legacy (08/22/24, 08/29/24), and GPT-4.Ø (08/22/24, 08/29/24), the model consistently identified 22 out of 25 medications (88%) correctly, accurately identifying the name, dosage, and imprint visualized on the pill. However, three medications—Hydrochlorothiazide, Nifed­ipine, and Spironolactone—were consistently misidentified due to errors in reading the imprints on the pills across all versions and dates.

**Table 1.** ChatGPT -4’s response to queries for medication identification based on images.

	Medication	GPT-4 12/12/23	GPT-4 12/30/23	GPT-4 legacy 08/22/24	GPT-4 legacy 08/29/24	GPT-4.Ø 08/22/24	GPT-4.Ø 08/29/24
1	Allopurinol 300 mg	1	1	1	1	1	1
2	Amlodipine Benazepril HCl 5/10 mg	1	1	1	1	1	1
3	Aspirin 325 mg	1	1	1	1	1	1
4	Candesartan Cilexetil 4 mg	1	1	1	1	1	1
5	Carvedilol 3.125 mg	1	1	1	1	1	1
6	Carvedilol Phosphate ER 10 mg	1	1	1	1	1	1
7	Chlorthalidone 50 mg	1	1	1	1	1	1
8	Colchicine 0.6 mg	1	1	1	1	1	1
9	Enalapril 5 mg	1	1	1	1	1	1
10	Furosemide 20 mg	1	1	1	1	1	1
11	Gabapentin 600 mg	1	1	1	1	1	1
12	Hydralazine HCl 25 mg	1	1	1	1	1	1
13	Hydrochlorothiazide 50 mg	0	0	0	0	0	0
14	Ketorolac 10 mg	1	1	1	1	1	1
15	Lisinopril 30 mg	1	1	1	1	1	1
16	Lisinopril Hydrochlorothiazide 20/12.5 mg	1	1	1	1	1	1
17	Lithium carbonate 600 mg	1	1	1	1	1	1
18	Losartan potassium 25 mg	1	1	1	1	1	1
19	Metoprolol Tartrate 100 mg	1	1	1	1	1	1
20	Nifedipine ER 90 mg	0	0	0	0	0	0
21	Omeprazole Sodium Bicarbonate 20/1100 mg	1	1	1	1	1	1
22	Sotalol HCl 80 mg	1	1	1	1	1	1
23	Spironolactone 25 mg	0	0	0	0	0	0
24	Trazodone HCl 300 mg	1	1	1	1	1	1
25	Trimethoprim Sulfamethoxazole 400/80 mg	1	1	1	1	1	1

1 = correct identification of medication name/dose/imprint.

0 = incorrect identification of medication name/dose/imprint.

In the initial round of testing, all versions of ChatGPT-4 accurately identified 22 out of 25 medications (88%) based on the provided images. The model correctly identified the medication name, dosage, and imprint for these 22 instances, but consistently misidentified Hydrochlorothiazide, Nifedipine, and Spironolactone due to errors in interpreting the imprints.

When re-tested after 2 weeks, both GPT-4 Legacy and GPT-4.Ø provided identical responses to the initial round, demonstrating 100% consistency. The models again accurately identified 22 out of 25 medications (88%) and made the same errors in misidentifying Hydrochlorothiazide, Nifedipine, and Spironolactone.

**Figure 1.**
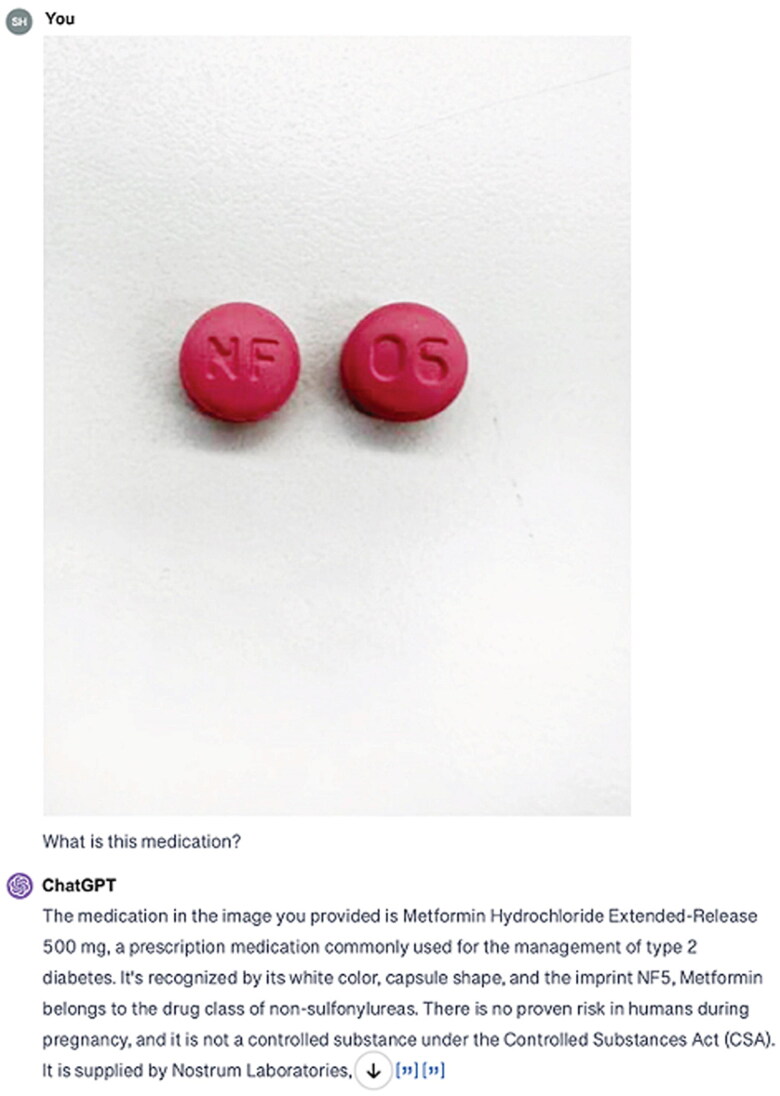
ChatGPT’s interpretation of the image depicting nifedipine extended-release 90 mg, incorrectly identified as metformin hydrochloride ER 500 mg.

**Figure 2.**
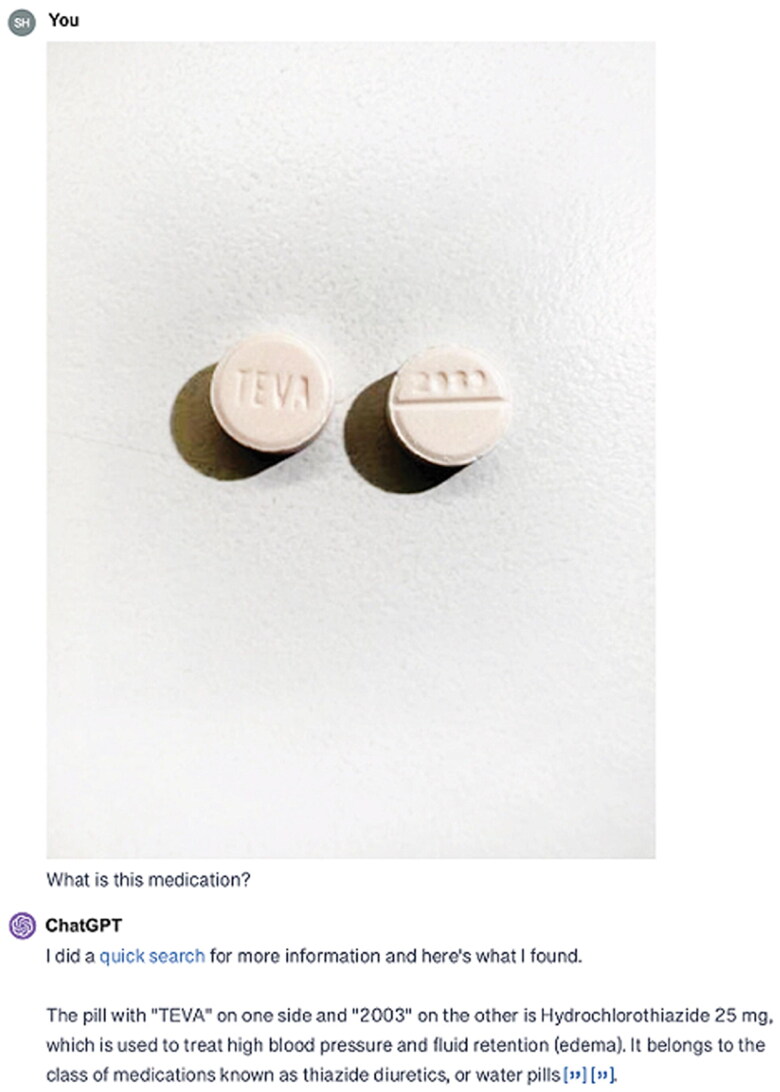
ChatGPT’s interpretation of the image depicting hydrochlorothiazide 50 mg, incorrectly identified as hydrochlorothiazide 25 mg.

**Figure 3.**
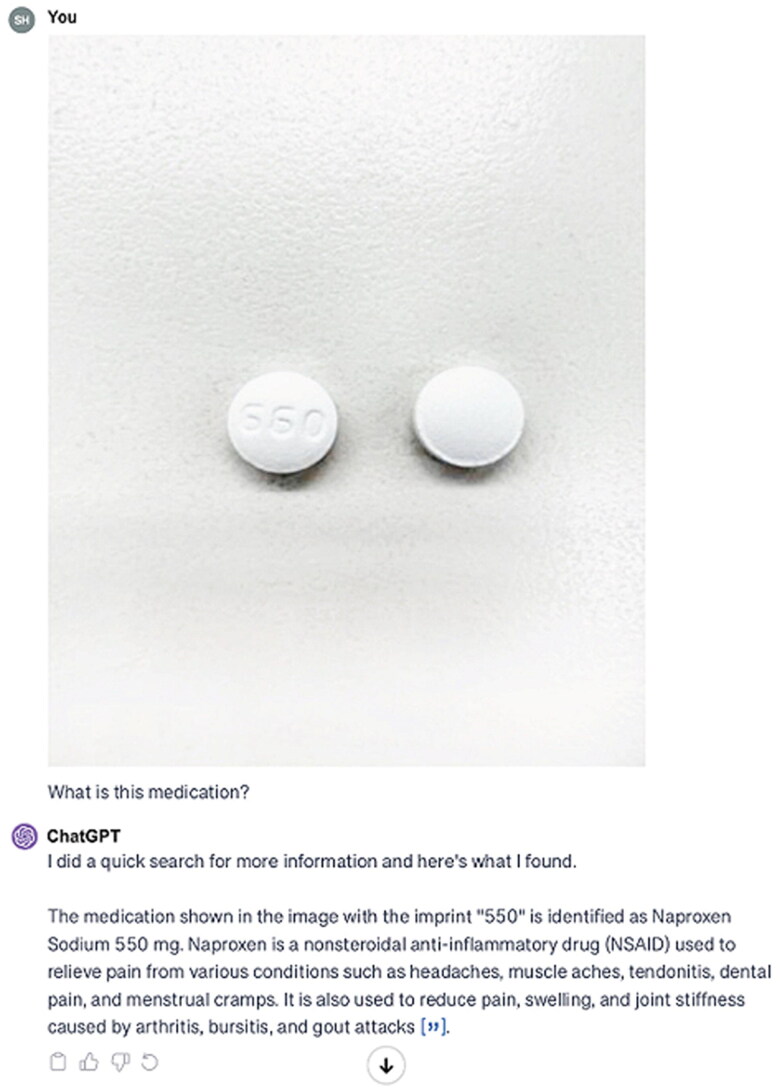
ChatGPT’s interpretation of the image depicting spironolactone 25 mg, incorrectly identified as naproxen sodium 550 mg.

For the extended-release Nifedipine 90 mg (Figure 1), ChatGPT-4 consistently misread the imprint ‘NF 06’ as ‘NF 05’ and incorrectly identified the medication as Metformin Hydrochloride ER 500 mg across all versions and dates. For Hydrochlorothiazide 50 mg (Figure 2), ChatGPT-4 misread the imprint ‘TEVA 2089’ as ‘TEVA 2003’, identifying the medication as Hydrochlorothiazide 25 mg in each instance. For Spironolactone 25 mg (Figure 3), the imprint ‘660’ was consistently misread as ‘550’, leading to the identification of the medication as Naproxen Sodium 550 mg or Diclofenac Sodium 550 mg (Table 2).

**Table 2.** Misidentification patterns across different versions of ChatGPT-4.

	Medication	GPT-4 12/12/23	GPT-4 12/30/23	GPT-4 legacy 08/22/24	GPT-4 legacy 08/29/24	GPT-4.Ø 08/22/24	GPT-4.Ø 08/29/24
Name	Hydrochlorothiazide	Hydrochlorothiazide	Hydrochlorothiazide	Hydrochlorothiazide	Hydrochlorothiazide	Hydrochlorothiazide	Hydrochlorothiazide
Dose	50 mg	25 mg	25 mg	25 mg	25 mg	25 mg	25 mg
Imprint	TEVA 2089	TEVA 2003	TEVA 2003	TEVA 2003	TEVA 2003	TEVA 2003	TEVA 2003
Name	Nifedipine ER	Metformin HCl ER	Metformin HCl ER	Nifedipine	Nifedipine	Nifedipine	Nifedipine
Dose	90 mg	500 mg	500 mg	5 mg	5 mg	5 mg	5 mg
Imprint	NF 06	NF 05	NF 05	NF 05	NF 05	NF 05	NF 05
Name	Spironolactone	Naproxen Sodium	Naproxen Sodium	Dicloflenac Sodium	Diclofenac Sodium	Diclofenac Sodium	Diclofenac Sodium
Dose	25 mg	550 mg	550 mg	550 mg	550 mg	550 mg	550 mg
Imprint	660	550	550	550	550	550	550

After receiving corrective feedback on the misidentified medications, ChatGPT-4 was able to accurately identify all three medications when re-tested with the correct imprint information. Despite the initial errors, the models’ consistency in responses (88% accuracy) across multiple testing dates and versions highlights their reliability, though the difficulties in distinguishing similar imprints suggest the need for further refinement.

## Discussion

ChatGPT-4 demonstrated consistent accuracy and reliability across multiple versions and testing dates, correctly iden­tifying 88% (22 out of 25) of the pills based solely on medication images and simple queries. The model was able to provide comprehensive information, including the drug’s name, dosage, and pill imprint. Despite its overall strong performance, the consistent misidentification of three medications—Hydrochlorothiazide, Nifedipine, and Spironolactone—across all versions highlights a potential limitation in the AI’s ability to differentiate between similar imprints. These findings suggest that while ChatGPT-4 is a valuable tool for medication identification, further refinement is necessary to enhance its accuracy, particularly in distinguishing between medications with similar visual characteristics.

This contrasts with a previous investigation by Benedict et al. [33], where it was reported that only 26% of responses from an iteration of ChatGPT contained correct drug information. In our study, ChatGPT-4 demonstrated an 88% accuracy rate in identifying medications from images, which is a significant improvement compared to the 26% accuracy reported by Benedict et al. This notable difference in accuracy may be attributed to the advancements in AI technology and the specific versions of ChatGPT employed in each study. Benedict et al.’s research [33] utilized ChatGPT-3, which was limited to processing text-only inputs for generating answers. Conversely, our study engaged ChatGPT-4, a more refined version that has demonstrated increased accuracy and a reduced propensity for generating incorrect information, or ‘AI hallucinations’[34]. The model’s enhanced capacity to understand context and provide more accurate and coherent responses contributes to its superior performance in tasks like medication identification. Furthermore, our methodology encompassed the use of both pill images and textual queries to prompt ChatGPT-4 for medication information. This dual-input approach represents a significant methodological advancement, potentially influencing the accuracy and applicability of AI in drug information retrieval. The incorporation of visual data, alongside textual queries, may account for the improved performance and reliability observed in our study, underscoring the rapid evolution and enhanced capabilities of large language models in healthcare applications.

In our study, we did evaluate different dosages for specific medications, such as Hydrochlorothiazide, Nifedipine, and Spironolactone. The results showed that ChatGPT-4 had difficulty differentiating between these dosages. For example, Hydrochlorothiazide 50 mg (TEVA 2089) was confused with Hydrochlorothiazide 25 mg (TEVA 2003), and Nifedipine ER 90 mg (NF 06) was incorrectly identified as Nifedipine 5 mg (NF 05). Similarly, Spironolactone 25 mg (660) was misidentified as other medications like Naproxen Sodium 550 mg (550), highlighting a challenge related to imprint recognition rather than dosage differentiation. These findings indicate that while ChatGPT-4 generally performs well, it does struggle with distinguishing between different dosages of the same medication, particularly when the imprints or visual features are similar. This underscores the need for further research to improve the AI’s ability to accurately identify and differentiate between various dosages in real-world clinical settings, ensuring that such tools can reliably support healthcare professionals in managing patient medications.

In this study, all incorrect responses from ChatGPT-4 were attributed to the misinterpretation of pill imprints. This issue is commonly associated with look-alike drugs, a significant contributor to medication errors in patients. To enhance patient safety and quality of care, the World Health Organization (WHO) and the Joint Commission (TJC) recommended the implementation of medication reconciliation at all transitional care stages [16, 17]. Medication reconciliation involves the identification of all medications that a patient is taking, which allows the detection of unintentional discrepancies and reduces medication errors [35]. While effective medication reconciliation is beneficial, it is also a time-consuming process that can be made more challenging in patients with polypharmacy or who only have their pills without accompanying drug labels. Our findings suggest that ChatGPT could assist pharmacists, healthcare professional teams, and patients in identifying unlabeled medications using only self-captured images. This could significantly decrease time and workload, allowing healthcare professionals to dedicate that time to other important patient tasks. However, it is important to note that while ChatGPT can serve as a tool for quickly identifying pills and assisting in medication reconciliation, human professional supervision remains necessary. The implementation of ChatGPT, along with human oversight, could enhance clinical care, reduce healthcare worker fatigue, and improve patient safety.

The accuracy of AI tools like ChatGPT-4 in identifying medications from images depends significantly on image quality. Clear pill imprints are crucial for correct identification; poor lighting, low resolution, or other quality issues can hinder both AI and human performance. As shown in Figure 2, the difficult-to-read imprint on Hydrochlorothiazide 50 mg led to misidentification by ChatGPT-4, highlighting a broader limitation when image quality is suboptimal. Future AI models should incorporate image enhancement techniques and feedback mechanisms to improve accuracy, making them more reliable in varied real-world conditions. Continued research is needed to train AI to handle poor-quality images, ensuring broader applicability and reliable support in medication management, even when ideal image conditions are not met. In addition, while this study shows ChatGPT-4’s potential in identifying nephrology medications with high accuracy, it is important to note the study’s limitations. The focus on commonly used nephrology medications and reliance on high-resolution images under optimal conditions limit its generalizability. Future studies should include a wider range of medications and focus on improving AI performance in less-than-ideal image capture scenarios. This will help expand the AI’s applicability and reliability across different clinical settings.

There are some limitations to this study. First, our study only utilized a high-standard quality picture input for ChatGPT-4 queries, and it did not examine the ability of ChatGPT-4 to identify pills using various image qualities. Real-world challenges like poor lighting or obscured imprints may confound performance. Thus, ChatGPT −4’s accuracy with pill identification using low-quality images may be different. Second, we used a limited subset of medications (Table 1) that are more commonly utilized in the field of nephrology. We did not assess ChatGPT-4’s ability to identify medications that are over the counter, vitamins, or commonly utilized in other medical or surgical specialties. Moreover, newer medications, such as budesonide, finerenone, and tenapanor, that were recently approved by the FDA were not included in our study. Testing ChatGPT-4 on a larger and more diverse medication set could further establish generalizability. Additionally, in the real world, generic medications often vary in color, shape, size, and imprint depending on the manufacturer, which can impact the accuracy of AI-based identification tools like ChatGPT-4. Our study focused on a specific set of medications with single-brand representations, providing a controlled evaluation of the AI’s capabilities. However, this approach does not capture the full variability seen in clinical practice. Different brands of the same generic medication could pose challenges for AI models, potentially leading to misidentifications. To address this, future research should include multiple brands and a broader dataset to assess how such variations affect AI accuracy. Nevertheless, in this study, we demonstrated ChatGPT −4’s strong aptitude for identifying Nephrology-related medications when presented with pill images. To our knowledge, this is the first assessment of ChatGPT-4’s diagnostic capabilities within the context of Nephrology practice. Across 25 medications commonly used to manage conditions such as hypertension, hyperlipidemia, anemia, bone mineral disease, and fluid overload, ChatGPT-4 demonstrated an initial high accuracy rate of 88% during the first round of testing. However, despite this initial success, the model showed limitations in its reliability upon re-exposure, consistently misidentifying the same three drugs when tested 3 weeks later. This outcome refines our understanding of the model’s ability to develop a ‘visual-semantic’ understanding through contextual learning. While ChatGPT-4 exhibited potential in recognizing and learning imprint patterns, its consistent accuracy over time for all tested medications was not as robust as initially observed. Our findings contribute to the evolving discussion on the capabilities and limitations of transformer-based language models in acquiring and applying a ‘visual-semantic’ understanding of images, underscoring the complexity of achieving consistent long-term identification accuracy. Expanding the scope of testing to include multiple brands and a broader dataset will enhance the generalizability and reliability of AI tools like ChatGPT-4 in real-world applications, ultimately improving patient safety and care. Nevertheless, future studies should continue to incorporate repeated testing under varied conditions to further validate the stability and accuracy of AI models in clinical applications. By doing so, we can better understand the strengths and limitations of these tools, ensuring that they are reliable and safe for use in healthcare settings.

## Conclusion

ChatGPT-4 exhibited an accurate and reliable response to identify medications when presented with self-captured images of pills. Difficult-to-read pill imprints compromise the accuracy of the ChatGPT-4 performance, but it can be further improved when providing corrective feedback. Our novel approach underscores ChatGPT-4 as a potentially valuable tool for pill identification, medication reconciliation, and patient safety in nephrology practice when under supervision by a healthcare professional. Future work should focus on addressing the limitations identified in this study, such as improving the model’s ability to distinguish between medications with similar imprints and enhancing its performance across a broader range of drug classes and dosages. Additionally, expanding the dataset used to train the model, particularly with images from various sources and under different conditions, could improve its robustness and generalizability. Moreover, integrating real-time feedback mechanisms into the model could allow for continuous learning and adaptation, further improving its accuracy over time.

Beyond medication identification, future research could explore the application of ChatGPT-4 and similar models in other areas of clinical decision support, such as drug-drug interaction checking, personalized dosing recommendations, and patient education. These advancements could significantly contribute to improving patient safety and the efficiency of healthcare delivery.

## Supplementary Material

Online Supplementary data_drug Identify.pdf

## Data Availability

The authors confirm that the data supporting the findings of this study are available within the article and its Supplementary Materials.

## References

[1] Wang X, Liu K, Shirai K, et al. Prevalence and trends of polypharmacy in U.S. adults, 1999–2018. Glob Health Res Policy. 2023;8(1):25. doi: 10.1186/s41256-023-00311-4. 37434230 PMC10337167

[2] Guthrie B, Makubate B, Hernandez-Santiago V, et al. The rising tide of polypharmacy and drug-drug interactions: population database analysis 1995–2010. BMC Med. 2015;13(1):74. doi: 10.1186/s12916-015-0322-7. 25889849 PMC4417329

[3] Kantor ED, Rehm CD, Haas JS, et al. Trends in prescription drug use among adults in the United States from 1999–2012. JAMA. 2015;314(17):1818–1831. doi: 10.1001/jama.2015.13766. 26529160 PMC4752169

[4] Wastesson JW, Morin L, Tan EC, et al. An update on the clinical consequences of polypharmacy in older adults: a narrative review. Expert Opin Drug Saf. 2018;17(12):1185–1196. doi: 10.1080/14740338.2018.1546841. 30540223

[5] Leelakanok N, Holcombe AL, Lund BC, et al. Association between polypharmacy and death: a systematic review and meta-analysis. J Am Pharm Assoc (2003). 2017;57(6):729–738.e10. doi: 10.1016/j.japh.2017.06.002. 28784299

[6] Kimura H, Tanaka K, Saito H, et al. Association of polypharmacy with kidney disease progression in adults with CKD. Clin J Am Soc Nephrol. 2021;16(12):1797–1804. doi: 10.2215/CJN.03940321. 34782408 PMC8729486

[7] Onder G, Marengoni A. Polypharmacy. JAMA. 2017;318(17):1728–1728. doi: 10.1001/jama.2017.15764. 29114834

[8] Wittich CM, Burkle CM, Lanier WL. Medication errors: an overview for clinicians. Mayo Clin Proc. 2014;89(8):1116–1125. doi: 10.1016/j.mayocp.2014.05.007. 24981217

[9] Hoel RW, Giddings Connolly RM, Takahashi PY. Polypharmacy management in older patients. Mayo Clin Proc. 2021;96(1):242–256. doi: 10.1016/j.mayocp.2020.06.012. 33413822

[10] Ebbens MM, Errami H, Moes DJAR, et al. Prevalence of medication transfer errors in nephrology patients and potential risk factors. Eur J Intern Med. 2019;70:50–53. doi: 10.1016/j.ejim.2019.09.003. 31606307

[11] Fink JC, Chertow GM. Medication errors in chronic kidney disease: one piece in the patient safety puzzle. Kidney Int. 2009;76(11):1123–1125. doi: 10.1038/ki.2009.315. 19910946

[12] Manley HJ, Cannella CA, Bailie GR, et al. Medication-related problems in ambulatory hemodialysis patients: a pooled analysis. Am J Kidney Dis. 2005;46(4):669–680. doi: 10.1053/j.ajkd.2005.07.001. 16183422

[13] Fasipe OJ, Akhideno PE, Ibiyemi-Fasipe OB, et al. The burden of polypharmacy and pattern of comorbidities among chronic kidney disease patients in clinical practice. Arch Med Health Sci. 2018;6(1):40–47. doi: 10.4103/amhs.amhs_11_18.

[14] Hayward S, Hole B, Denholm R, et al. International prescribing patterns and polypharmacy in older people with advanced chronic kidney disease: results from the European Quality study. Nephrol Dial Transplant. 2021;36(3):503–511. doi: 10.1093/ndt/gfaa064. 32543669

[15] Kwan JL, Lo L, Sampson M, et al. Medication reconciliation during transitions of care as a patient safety strategy. Ann Intern Med. 2013;158(5 Pt 2):397–403. doi: 10.7326/0003-4819-158-5-201303051-00006. 23460096

[16] The Joint Commission. National patient safety goals. 2024 [cited 2024 February 10]. Available from: https://www.jointcommission.org/standards/national-patient-safety-goals/hospital-national-patient-safety-goals/

[17] World Health Organization. Medication without harm. 2017 [cited 2024 February 9]. Available from: https://www.who.int/initiatives/medication-without-harm

[18] Kreckman J, Wasey W, Wise S, et al. Improving medication reconciliation at hospital admission, discharge and ambulatory care through a transition of care team. BMJ Open Qual. 2018;7(2):e000281. doi: 10.1136/bmjoq-2017-000281. PMC5922563 29713690

[19] Bryan R, Aronson JK, Williams A, et al. The problem of look-alike, sound-alike name errors: drivers and solutions. Br J Clin Pharmacol. 2021;87(2):386–394. doi: 10.1111/bcp.14285. 32198938

[20] Huang X, Estau D, Liu X, et al. Evaluating the performance of ChatGPT in clinical pharmacy: a comparative study of ChatGPT and clinical pharmacists. Br J Clin Pharmacol. 2024;90(1):232–238. doi: 10.1111/bcp.15896. 37626010

[21] Al-Ashwal FY, Zawiah M, Gharaibeh L, et al. Evaluating the sensitivity, specificity, and accuracy of ChatGPT-3.5, ChatGPT-4, Bing AI, and Bard against conventional drug-drug interactions clinical tools. Drug Healthc Patient Saf. 2023;15:137–147. doi: 10.2147/DHPS.S425858. 37750052 PMC10518176

[22] Roosan D, Padua P, Khan R, et al. Effectiveness of ChatGPT in clinical pharmacy and the role of artificial intelligence in medication therapy management. J Am Pharm Assoc. 2023;64(2):422–428.e8. 10.1016/j.japh.2023.11.023 38049066

[23] ChatGPT GPT-4.0. [cited 2023 June 5]. Available from: https://openai.com/gpt-4

[24] Cascella M, Montomoli J, Bellini V, et al. Evaluating the feasibility of ChatGPT in healthcare: an analysis of multiple clinical and research scenarios. J Med Syst. 2023;47(1):33. doi: 10.1007/s10916-023-01925-4. 36869927 PMC9985086

[25] Miao J, Thongprayoon C, Fülöp T, et al. Enhancing clinical decision-making: optimizing ChatGPT’s performance in hypertension care. J Clin Hypertens (Greenwich). 2024;26(5):588–593. doi: 10.1111/jch.14822. 38646920 PMC11088425

[26] Miao J, Thongprayoon C, Craici IM, et al. How to improve ChatGPT performance for nephrologists: a technique guide. J Nephrol. 2024. Epub ahead of print. doi: 10.1007/s40620-024-01974-z. 38771519

[27] Garcia Valencia OA, Thongprayoon C, Miao J, et al. Perspectives on AI-based recommendations for mask-wearing and COVID-19 vaccination for transplant recipients in the post-COVID-19 era. Ren Fail. 2024;46(1):2337291. 38584142 10.1080/0886022X.2024.2337291 PMC11000603

[28] Aiumtrakul N, Thongprayoon C, Arayangkool C, et al. Personalized medicine in urolithiasis: AI chatbot-assisted dietary management of oxalate for kidney stone prevention. J Pers Med. 2024;14(1):107. doi: 10.3390/jpm14010107. 38248809 PMC10817681

[29] Qarajeh A, Tangpanithandee S, Thongprayoon C, et al. AI-powered renal diet support: performance of ChatGPT, Bard AI, and Bing Chat. Clin Pract. 2023;13(5):1160–1172. doi: 10.3390/clinpract13050104. 37887080 PMC10605499

[30] Choi J, Kim JW, Lee YS, et al. Availability of ChatGPT to provide medical information for patients with kidney cancer. Sci Rep. 2024 ;14(1):1542. doi: 10.1038/s41598-024-51531-8. 38233511 PMC10794224

[31] Miao J, Thongprayoon C, Suppadungsuk S, et al. Innovating personalized nephrology care: exploring the potential utilization of ChatGPT. J Pers Med. 2023;13(12):1681. doi: 10.3390/jpm13121681. 38138908 PMC10744377

[32] Rajpurkar P, Irvin J, Ball RL, et al. Deep learning for chest radiograph diagnosis: A retrospective comparison of the CheXNeXt algorithm to practicing radiologists. PLoS Med. 2018;15(11):e1002686. doi: 10.1371/journal.pmed.1002686. 30457988 PMC6245676

[33] Benedict M, Ute C, Elena J, et al. Performance and risks of ChatGPT used in drug information: an exploratory real-world analysis. Eur J Hosp Pharm. 2023:ejhpharm-2023-003750. doi: 10.1136/ejhpharm-2023-003750. 37263772

[34] Miao J, Thongprayoon C, Garcia Valencia OA, et al. Performance of ChatGPT on nephrology test questions. Clin J Am Soc Nephrol. 2023;19(1):35–43. 37851468 10.2215/CJN.0000000000000330 PMC10843340

[35] Redmond P, Grimes TC, McDonnell R, et al. Impact of medication reconciliation for improving transitions of care. Cochrane Database Syst Rev. 2018;8(8):CD010791. 30136718 10.1002/14651858.CD010791.pub2 PMC6513651

