# Supplementary material for: Identification of kidney-related medications using AI from self-captured pill images
Source: Ren Fail. 2024 Sep 11;46(2):2402075. doi: 10.1080/0886022X.2024.2402075 (PMC11391868; doi:10.1080/0886022X.2024.2402075)
Supplement: Online Supplementary data_drug Identify.pdf [file IRNF_A_2402075_SM8674.pdf]

1. Lisinopril 30 mg

You

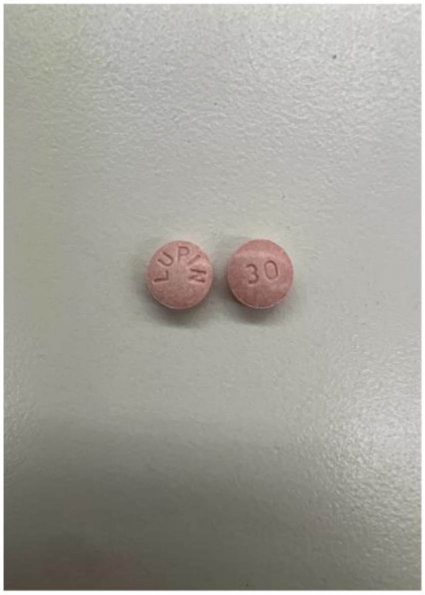

What is this medication?

ChatGPT

The pill shown with "LUPIN" on one side and "30" on the other is Lisinopril 30 mg, manufactured by Lupin Pharmaceuticals. Lisinopril is used to treat high blood pressure (hypertension), heart failure, and to improve survival after a heart attack.

2. Lithium Carbonate 600 mg

You

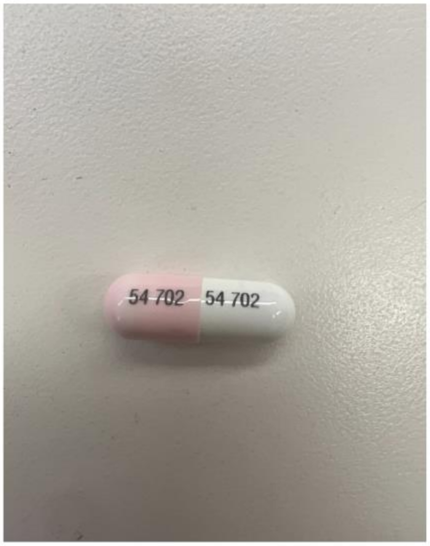

What is this medication?

ChatGPT

Based on a quick search, here's what I found.

The medication with the imprint "54 702" on a pink and white capsule is Lithium Carbonate 600 mg, which is supplied by Hikma Pharmaceuticals PLC. Lithium Carbonate is commonly used in the treatment of Bipolar Disorder, Schizoaffective Disorder, and Mania and is classified as an antimanic agent. There is evidence of human fetal risk if taken during pregnancy.

3. Lisinopril HCTZ 20/12.5 mg

You

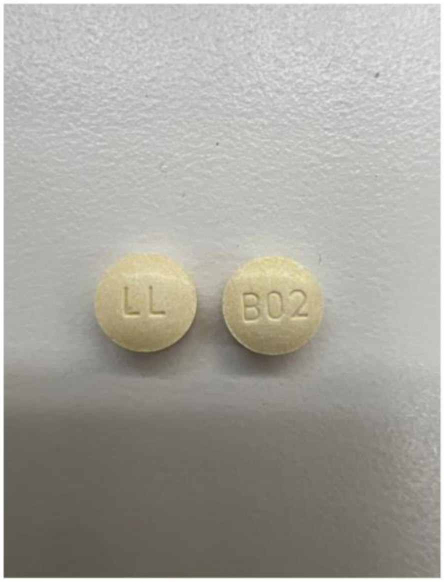

What is this medication?

ChatGPT

The pill with the imprint "LL" on one side and "B02" on the other is a combination of Hydrochlorothiazide 12.5 mg and Lisinopril 20 mg, manufactured by Lupin Pharmaceuticals, Inc. This medication is commonly used to treat high blood pressure (hypertension) [↑].

4. Losartan Potassium 25 mg

You

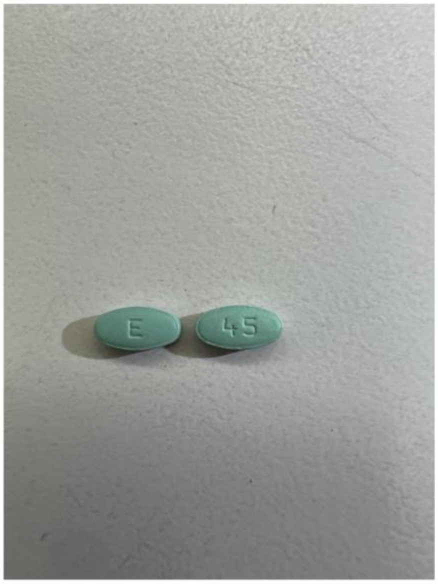

What is this medication?

ChatGPT

The medication with the imprint "E 45" is Losartan Potassium 25 mg. It is supplied by Aurobindo Pharma and is used in the treatment of high blood pressure, diabetic kidney disease, and is part of the drug class angiotensin receptor blockers. There is positive evidence of human fetal risk during pregnancy if taken without proper medical advice [↑].

5. Chlorthalidone 50 mg

You

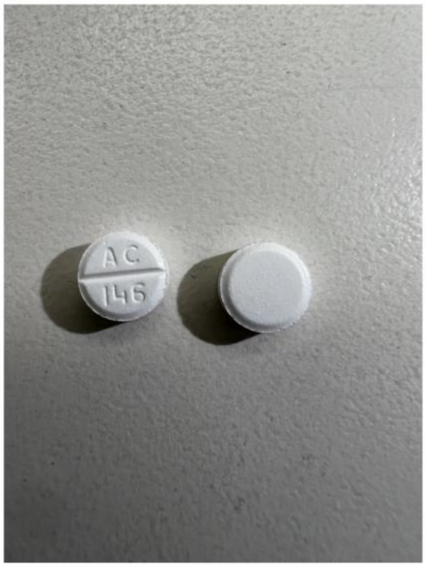

What is this medication?

ChatGPT

The pill with the imprint "AC 146" is identified as Chlorthalidone 50 mg. It is a prescription medication used to treat high blood pressure (hypertension) and edema. Chlorthalidone is a thiazide diuretic (water pill) that helps prevent your body from absorbing too much salt, which can cause fluid retention [↑] [↑].

6. Hydrochlorothiazide 50 mg

You

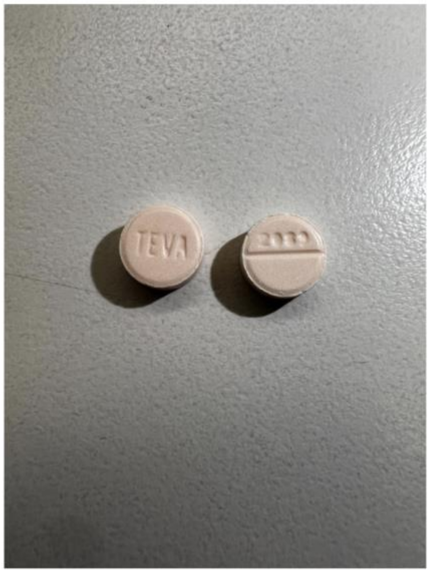

What is this medication?

ChatGPT

I did a quick search for more information and here's what I found.

The pill with "TEVA" on one side and "2003" on the other is Hydrochlorothiazide 25 mg, which is used to treat high blood pressure and fluid retention (edema). It belongs to the class of medications known as thiazide diuretics, or water pills [↑] [↑].

7. Hydralazine HCl 25 mg

You

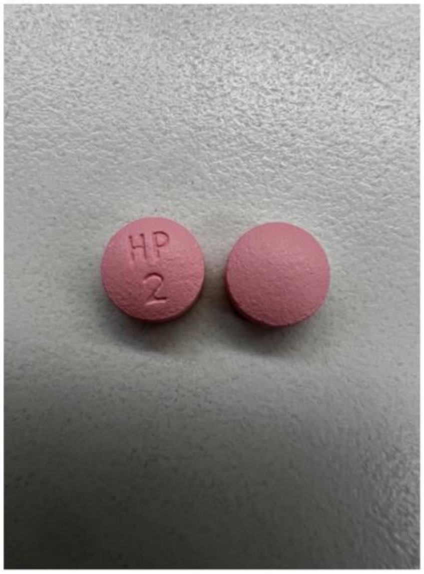

What is this medication?

ChatGPT

The pill with "HP" on one side and "2" on the other is identified as Hydralazine Hydrochloride 25 mg. It is used to treat high blood pressure (hypertension) and is also sometimes used to treat heart failure. Hydralazine belongs to a class of medications called vasodilators, which work by relaxing the blood vessels so that blood can flow more easily [↑].

8. Gabapentin 600 mg

You

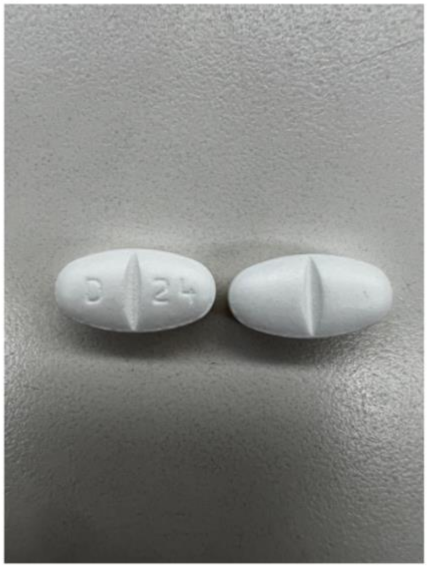

What is this medication?

ChatGPT

The medication shown in the image, marked with "D 24" on one side, is Gabapentin 600 mg. It is prescribed for the treatment of nerve pain associated with shingles (postherpetic neuralgia), for seizures in adults and children who have epilepsy, and sometimes for the treatment of restless legs syndrome (RLS). Gabapentin is in the class of drugs known as anticonvulsants or antiepileptic drugs.

9. Enalapril 5 mg

You

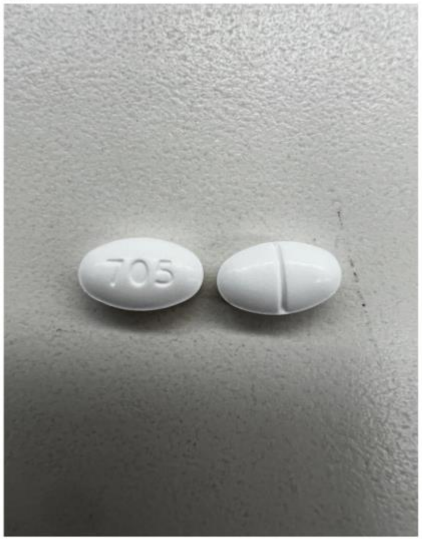

What is this medication?

ChatGPT

I did a quick search for more information and here's what I found.

The white oval pill with the imprint "705" is identified as Enalapril Maleate 5 mg. It is supplied by Avet Pharmaceuticals Inc. and is used in the treatment of high blood pressure, heart failure, and other conditions such as Alport Syndrome and diabetic kidney disease. Enalapril belongs to the drug class of Angiotensin-Converting Enzyme (ACE) Inhibitors [”].

10. Ketorolac 10 mg

You

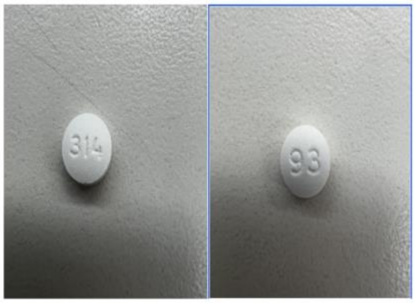

What is this medication?

ChatGPT

From a quick search, here's what I discovered.

The medication shown in the image with the imprints "314" on one side and "93" on the other is Ketorolac Tromethamine 10 mg. It is a nonsteroidal anti-inflammatory drug (NSAID) used for the short-term treatment of moderate to severe pain in adults. It is commonly prescribed for pain relief after surgery. Ketorolac is the generic name, and it's also known by the brand name Toradol. It works by blocking your body's production of certain natural substances that cause inflammation, which helps to decrease swelling, pain, or fever [”] [”].

11. Spironolactone 25 mg

You

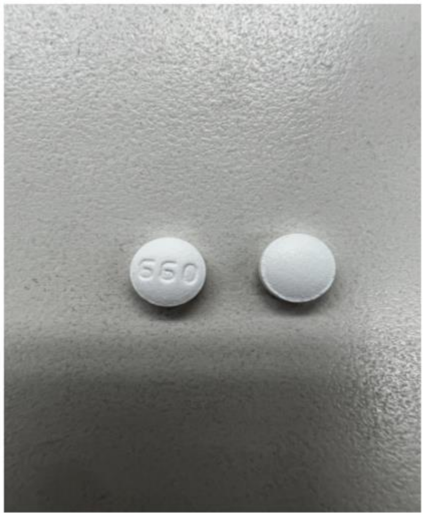

What is this medication?

ChatGPT

I did a quick search for more information and here's what I found.

The medication shown in the image with the imprint "550" is identified as Naproxen Sodium 550 mg. Naproxen is a nonsteroidal anti-inflammatory drug (NSAID) used to relieve pain from various conditions such as headaches, muscle aches, tendonitis, dental pain, and menstrual cramps. It is also used to reduce pain, swelling, and joint stiffness caused by arthritis, bursitis, and gout attacks [”].

12. Aspirin 325 mg

You

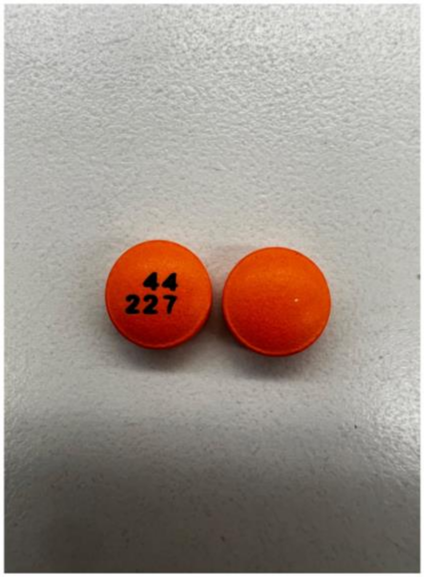

What is this medication?

ChatGPT

The medication depicted with "44 227" on it is Aspirin 325 mg. It is a nonsteroidal anti-inflammatory drug (NSAID) used to treat pain, fever, and inflammation. It is also used as an antiplatelet agent to prevent blood clots in patients at risk for heart attack or stroke [”] [”].

13. Carvedilol 3.125 mg

You

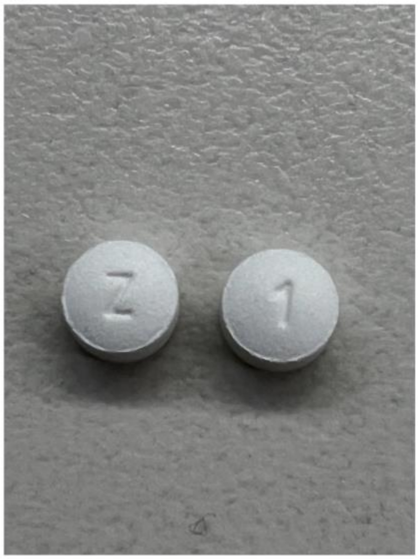

What is this medication?

ChatGPT

I did a quick search and here's what I found.

The white round pill with "Z" on one side and "1" on the other is Carvedilol 3.125 mg. It is a non-cardioselective beta blocker used in the treatment of high blood pressure, heart failure, left ventricular dysfunction, and sometimes angina. Carvedilol is supplied by Zydus Pharmaceuticals [״].

14. Carvedilol phosphate ER 10 mg

You

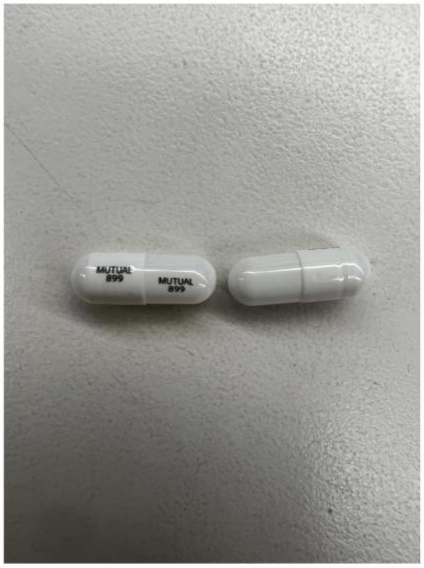

What is this medication?

ChatGPT

I did a quick search for more information and here's what I discovered.

The medication shown in the image, marked with "MUTUAL 899" on a white capsule, is Carvedilol Phosphate Extended-Release 10 mg. It is used for treating high blood pressure and heart failure and is part of a class of drugs called beta-blockers [״].

15. Allopurinol 300 mg

You

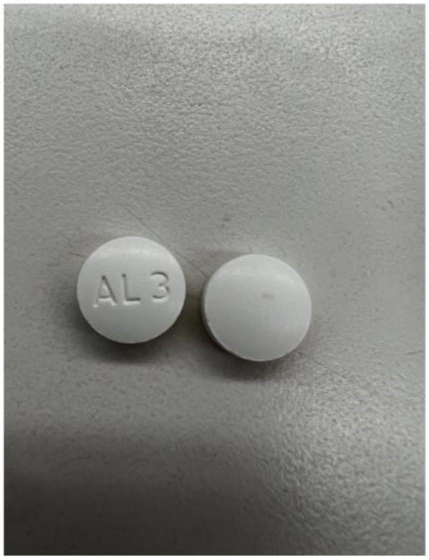

What is this medication?

ChatGPT

The white round pill with the imprint "AL3" is identified as Allopurinol 300 mg. Allopurinol is used in the treatment of gout, calcium oxalate calculi with hyperuricosuria (a type of kidney stone), and hyperuricemia secondary to chemotherapy. It belongs to the drug classes of antigout agents and antihyperuricemic agents [״].

16. Candesartan Cilexetil 4 mg

You

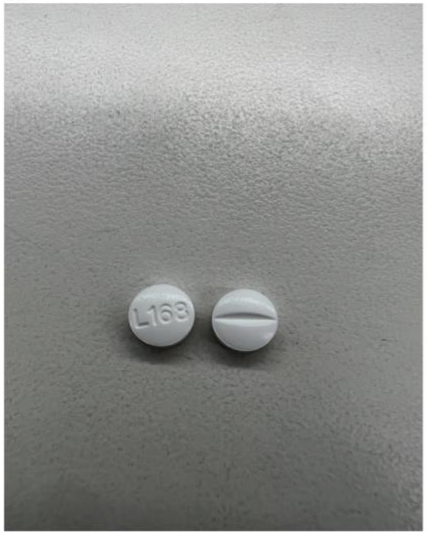

What is this medication?

ChatGPT

I did a quick search for more information and here's what I found.

The white round pill with the imprint "L168" is identified as Candesartan Cilexetil 4 mg, which is an angiotensin receptor blocker used for treating high blood pressure and heart failure. There is positive evidence of human fetal risk if used during pregnancy, so it should only be used when clearly needed and prescribed by a healthcare provider [״].

17. Omeprazole NaHCO3

You

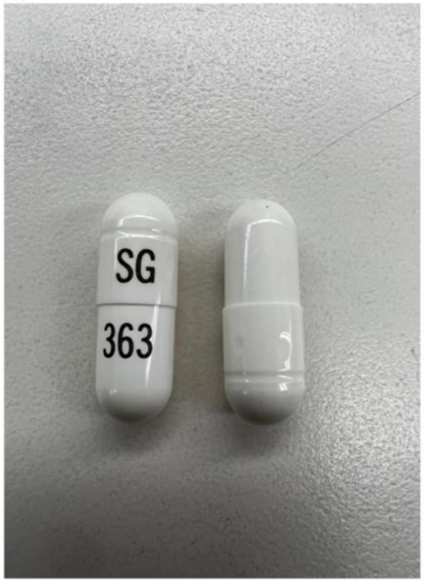

what is this medication?

ChatGPT

The capsule with "SG 363" on it is Omeprazole and Sodium Bicarbonate, containing 20 mg of omeprazole and 1100 mg of sodium bicarbonate. It is used for the treatment of conditions such as duodenal ulcer, erosive esophagitis, gastrointestinal hemorrhage, GERD, stomach ulcer, and other related conditions [↗].

18. Furosemide 20 mg

ChatGPT 4

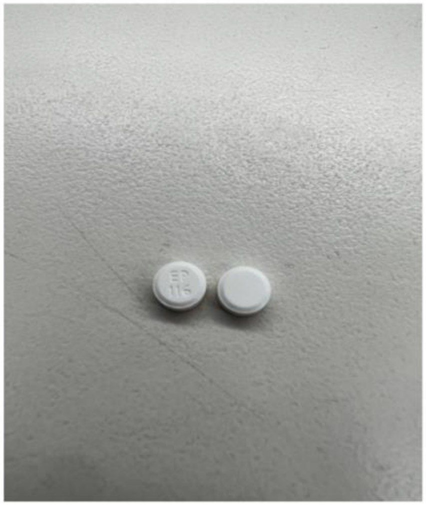

what is this medication?

ChatGPT

I did a quick search and here's what I discovered.

The white round pill with the imprint "EP 116" is identified as Furosemide 20 mg. Furosemide is a loop diuretic (water pill) that prevents your body from absorbing too much salt, allowing the salt to instead be passed in your urine. It is used to treat fluid retention (edema) in people with congestive heart failure, liver disease, or a kidney disorder such as nephrotic syndrome. This medication is also used to treat high blood pressure (hypertension) [↗] [↗].

19. Trazodone HCl 300 mg

You

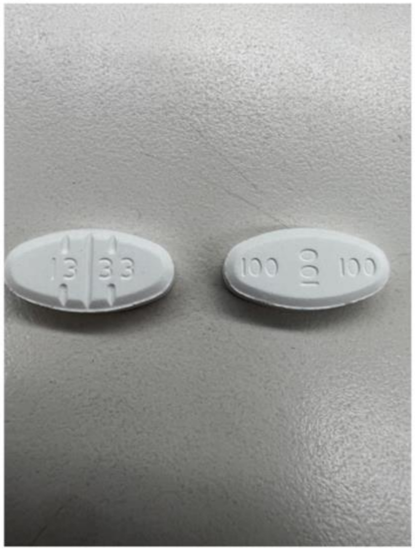

what is this medication?

ChatGPT

From a quick search, here's what I discovered.

The medication in the image you provided is Trazodone Hydrochloride 300 mg, used in the treatment of depression, major depressive disorder, and for sedation. It is categorized under the drug class of phenylpiperazine antidepressants. This particular medication should be used under prescription and the risk cannot be ruled out during pregnancy [↗].

20. Metoprolol tartrate 100 mg

You

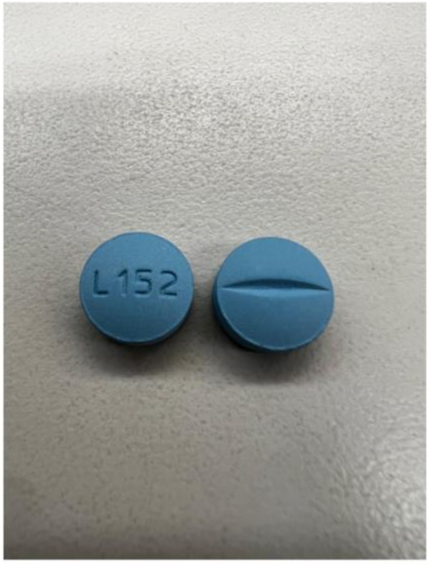

what is this medication?

ChatGPT

The medication in the image you provided is Metoprolol Tartrate 100 mg, which is a blue, round pill with the imprint L152. It's supplied by Alembic Pharmaceuticals Limited and is used to treat conditions such as angina, high blood pressure, heart failure, and to prevent heart attacks. Metoprolol belongs to the cardioselective beta blockers drug class. It is a prescription-only medication and the risk cannot be ruled out during pregnancy [↗].

21. Nifedipine ER 90 mg

You

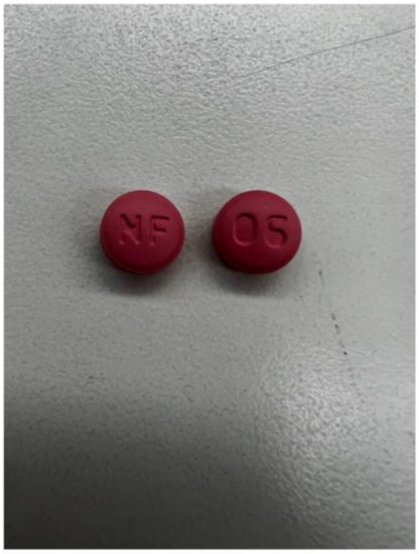

What is this medication?

ChatGPT

The medication in the image you provided is Metformin Hydrochloride Extended-Release 500 mg, a prescription medication commonly used for the management of type 2 diabetes. It's recognized by its white color, capsule shape, and the imprint NF5. Metformin belongs to the drug class of non-sulfonylureas. There is no proven risk in humans during pregnancy, and it is not a controlled substance under the Controlled Substances Act (CSA). It is supplied by Nostrum Laboratories, [*] [*].

22. Amlodipine/Benazepril 5/10 mg

You

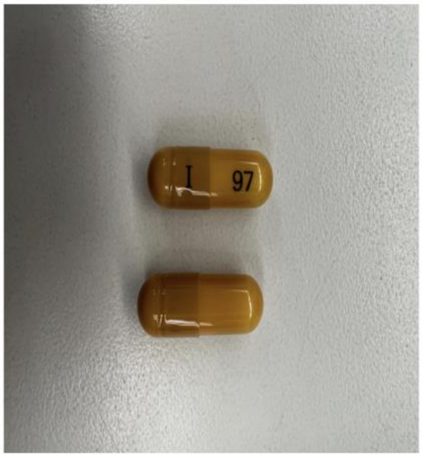

What is this medication?

ChatGPT

The medication shown is Amlodipine Besylate and Benazepril Hydrochloride 5 mg / 10 mg, which is used for the treatment of high blood pressure. It's a combination drug that includes a calcium channel blocker (Amlodipine) and an ACE inhibitor (Benazepril). It's important to take it as prescribed by a healthcare provider.

23. Colchicine 0.6 mg

You

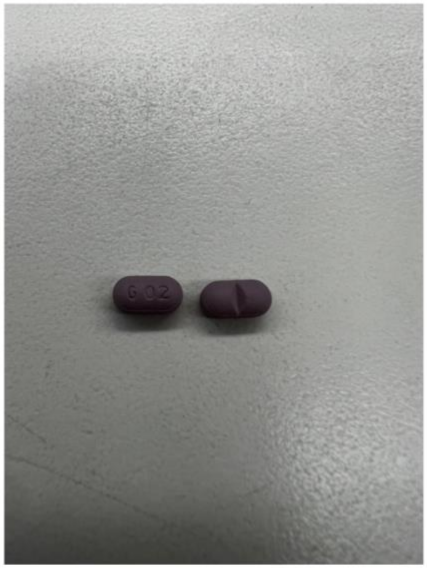

What is this medication?

ChatGPT

The medication shown in the image with the imprint "G 02" is Colchicine 0.6 mg, which is purple and capsule-shaped. Colchicine is typically used to prevent gout flares and to treat Familial Mediterranean Fever. It is categorized under the drug class of antigout agents. While it is not a controlled substance, there is a risk associated with its use during pregnancy, so caution is advised [*].

24. Bactrim 400/80 mg

You

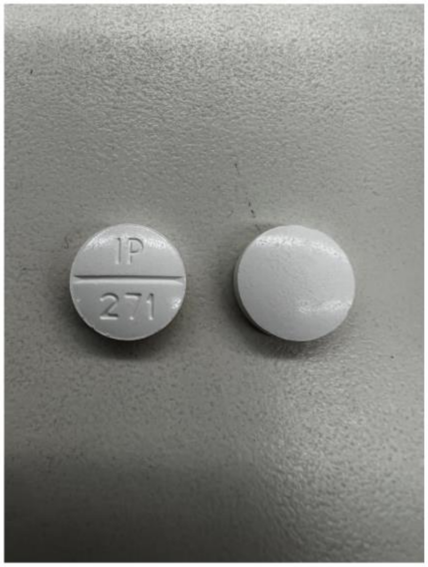

what is this medication?

ChatGPT

The medication in the image with the imprint "IP 271" is Sulfamethoxazole and Trimethoprim 400 mg / 80 mg. This combination is used to treat bacterial infections and is part of the sulfonamides drug class. While it is not a controlled substance, there is a risk associated with its use during pregnancy. It is supplied by Amneal Pharmaceuticals [*].

25. Sotalol HCl 80mg

ChatGPT 4

SH **You**

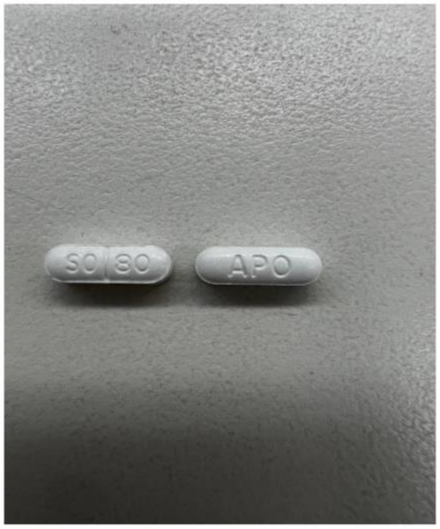

What is this medication?

ChatGPT

I did a quick search for more information and here's what I discovered.

The medication with the imprint "SO 180" on one side and "APO" on the other is Sotalol Hydrochloride 80 mg. It's a white oval pill used to treat conditions like atrial fibrillation, atrial flutter, and ventricular arrhythmia. Sotalol belongs to the group III antiarrhythmics and non-cardioselective beta blockers drug classes. It's regarded as safe during pregnancy with no proven risk in humans and is not a controlled substance [”].
